# Crimean-Congo Hemorrhagic Fever Virus in Ticks Collected from Cattle, Corsica, France, 2023

**DOI:** 10.3201/eid3005.231742

**Published:** 2024-05

**Authors:** Paloma Kiwan, Shirley Masse, Geraldine Piorkowski, Nazli Ayhan, Morena Gasparine, Laurence Vial, Remi N. Charrel, Xavier de Lamballerie, Alessandra Falchi

**Affiliations:** Unité des Virus Emergents, Aix Marseille Université, Università di Corsica, IRD140, INSERM 207 IRBA, Marseille, France (P. Kiwan, S. Masse, G. Piorkowski, N. Ayhan, M. Gasparine, R.N. Charrel, X. de Lamballerie, A. Falchi);; Université de Corse–Institut National de Santé et de la Recherche Médicale, Corte, France (P. Kiwan, S. Masse, G. Piorkowski, N. Ayhan, M. Gasparine, R.N. Charrel, X. de Lamballerie, A. Falchi);; Centre National de Référence des Arbovirus, Marseille, France (N. Ayhan, X. de Lamballerie);; Université de Montpellier, Montpellier, France (L. Vial)

**Keywords:** Crimean-Congo hemorrhagic fever virus, Crimean-Congo hemorrhagic fever, viruses, vector-borne infections, tickborne infections, ticks, cattle, Corsica, France, zoonoses

## Abstract

We report the detection of Crimean-Congo hemorrhagic fever virus (CCHFV) in Corsica, France. We identified CCHFV African genotype I in ticks collected from cattle at 2 different sites in southeastern and central-western Corsica, indicating an established CCHFV circulation. Healthcare professionals and at-risk groups should be alerted to CCHFV circulation in Corsica.

Crimean-Congo hemorrhagic fever (CCHF) is a tickborne disease caused by CCHF virus (CCHFV) (species *Orthonairovirus haemorrhagiae*, genus *Orthonairovirus,* family *Nairoviridae*, order *Bunyavirales*). Endemic in Africa, the Middle East, Asia, and Eastern Europe, CCHF has expanded to Western Europe (*1*). Repeated detection of CCHFV in Spain (*2*) raises questions about its circulation in neighboring countries, such as Portugal, Italy, and France.

In Corsica, a French Mediterranean island, a seroprevalence study of CCHFV conducted in livestock (cattle, goats, and sheep) during 2014–2016 showed an overall seroprevalence of 9.1%, and cattle harbored the highest rates (*3*). A subsequent surveillance study of 8,051 ticks collected from wild (wild boar, deer, and mouflon sheep) and domestic (cattle, horses, sheep) animals during 2016–2020 failed to detect CCHFV or nairovirus RNA (*4*).

Since 2022, we have continued CCHFV surveillance by collecting ticks from cattle at 2 slaughterhouses >2 times/month. Cattle originate from a broad geographic area, and the national ear-tag identification system enables tracing of each animal’s origin and farm owner (Figure). We identified ticks by using taxonomic keys, then pooled ticks by species, sex, development stage, study site, and animal host, as previously reported (*4*). We spiked each pool, consisting of 1–6 ticks, with a predefined amount of MS2 bacteriophage for monitoring nucleic acid extraction, reverse transcription PCR (RT-PCR), and nucleic acid amplification (*5*). We used MagMAX Viral/Pathogen Ultra Nucleic Acid Isolation Kit (Thermo Fisher Scientific, https://www.thermofisher.com) to purify nucleic acids. We tested each sample by using 2 real-time RT-PCRs, 1 targeting the large (L) RNA segment (*2*) and 1 targeting the small (S) RNA segment (*6*). We used the SuperScript IV One-Step RT-PCR System Kit (ThermoFisher) to design 28 CCHFV-specific pairs of primers to amplify the S, medium (M), and L segments (Appendix). We sequenced PCR products by using S5 Ion Torrent technology (ThermoFisher). We determined the best model by using the maximum-likelihood method and performed phylogenetic analyses by using MEGA6 software (*7*) (Figure).

**Figure:**
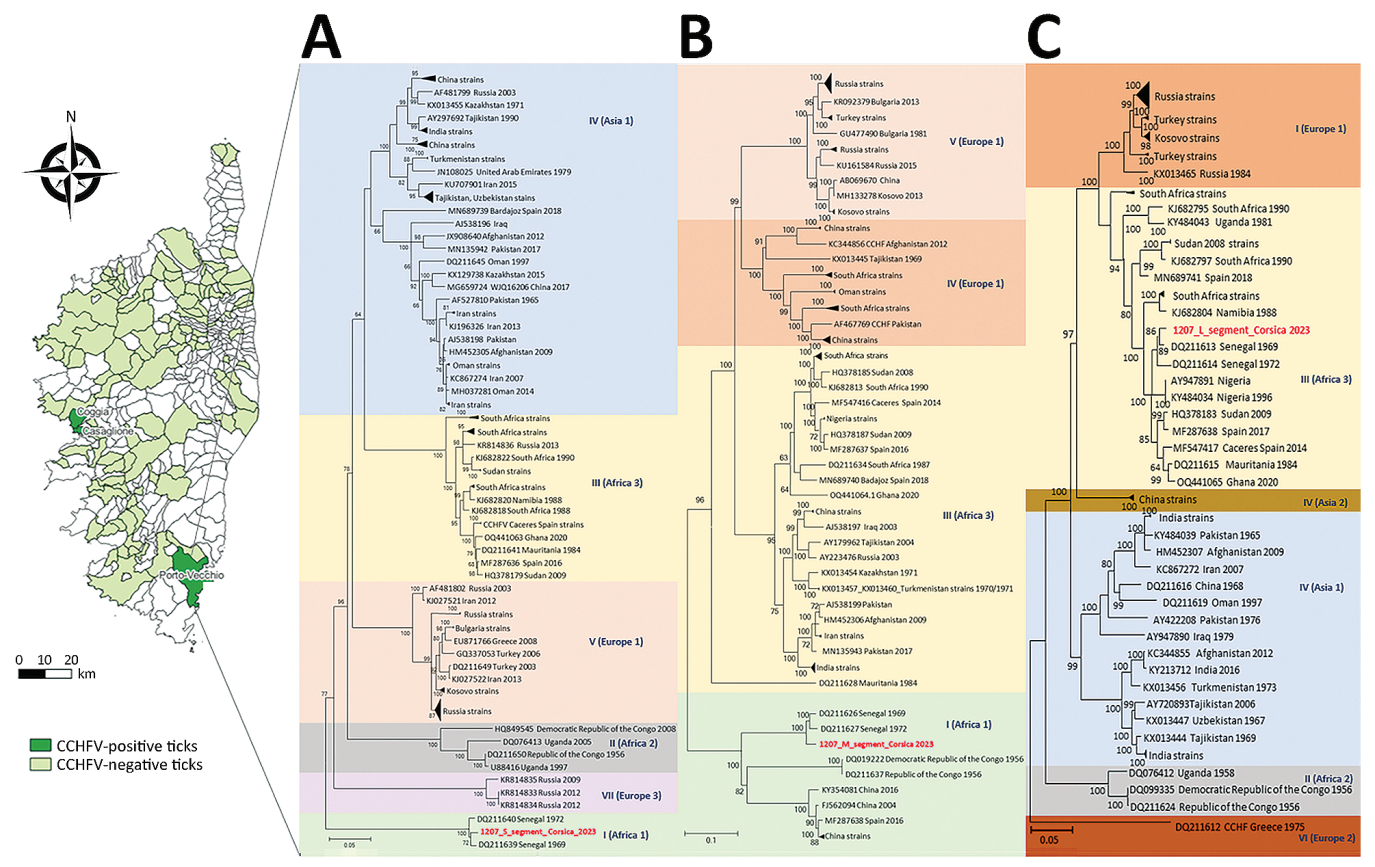
Phylogenetic analysis of Crimean-Congo hemorrhagic fever virus in ticks collected from cattle, Corsica, France, 2023. Map at left shows locations of cattle from which ticks were collected at the slaughterhouses of Ponte Leccia in the north and Cuttoli-Cortichiatto in the south during 2022­–2023. Phylogenetic trees show small (A), medium (B), and large (C) RNA segments CCHFV strains. Red font indicates strains detected from Corsica; other sequences are named by GenBank accession number, geographic origin, and sampling year. Evolutionary analyses were conducted in MEGA6 (https://www.megasoftware.net) after best model determination. The optimal tree is shown for each fragment. Trees were constructed using the maximum-likelihood method based on sequences on the small (Tamura-Nei model), medium (general time-reversible model), and large (Tamura-Nei model) segments of the virus. All positions with <95% sequence site coverage were eliminated (i.e., <5% alignment gaps, missing data, or ambiguous bases were allowed at any position [partial deletion option]). Results of bootstrap test (1,000 replicates) are shown next to the branches. Genotypes are indicated by Roman numerals (*8*) with the equivalent clade nomenclature (*9*): I, West Africa (Africa 1); II, Central Africa (Africa 2); III, South and West Africa (Africa 3); IV, Middle East/Asia, divided into 2 groups Asia 1 and Asia 2; V (Europe 1), Europe/Turkey (Europe 1); VI, Greece (Europe 2); VII (Europe 3). Scale bars indicate nucleotide substitutions per site. CCHFV, Crimean-Congo hemorrhagic fever virus; L-RNA, large segment of CCHFV RNA.

During June 2022–July 2023, we collected 5,165 ticks from 465 cattle and grouped ticks into 1,491 pools. Tick species consisted of 2,390 (46.27%) *Rhipicephalus bursa*, 1,103 (21.35%) *Hyalomma marginatum*, 750 (14.52%) *Boophilus annulatus*, 507 (9.81%) *Hyalomma scupense*, 238 (4.60%) *Haemaphysalis punctata*, 127 (2.45%) *Ixodes ricinus*, 48 (0.92%) *Rhipicephalus sanguineus*, and 2 (0.03%) *Dermacentor marginatus*. A total of 24 (1.70%) pools collected from 5 cattle from southern Corsica tested positive by the L-RNA assay (Table). Nineteen of the 24 tick pools were collected from 1 animal (no. 4039) (Table). Partial sequences for S (1,340 bp), M (4,894 bp), and L (11,275 bp) segments were obtained from animal nos. 2478 (pool 417) and 4039 (pool 1207) (Table). The effective detection of CCHFV genome is strongly supported by the formal exclusion of contamination because no CCHFV strain or genome had been previously processed in the laboratory, the PCR systems used can distinguish genomic RNA from the positive control (*6*), and the CCHFV sequences obtained were original and unambiguous.

**Table:** Description of Crimean-Congo hemorrhagic fever virus in tick pools collected from cattle, Corsica, France, 2023*

Cattle ID nos.	Cattle origin	Collection date	CCHFV–positive pool nos.	CCHFV assay, Ct		Tick species	Tick sex	No. ticks per pool
				L segment	S segment			
2478	Coggia	2022 Sep 27	417	IND	IND	*Haemaphysalis punctata*	F	1
6069	Coggia	2022 Sep 27	418	IND	IND	*H. punctata*	M	1
4376	Casaglione	2023 May 9	1252	IND	IND	*Rhipicephalus bursa*	M	6
			1253	IND	IND	*R. bursa*	M	6
4371	Casaglione	2023 May 9	1233	40.8	IND	*Hyalomma marginatum*	M	6
4039	Porto-Vecchio	2023 May 9	1204	35.9	IND	*R. bursa*	M	6
			1205	34.7	IND	*R. bursa*	F	6
			1206	34	IND	*R. bursa*	F	6
			1207	31.9	40	*R. bursa*	M	6
			1208	34.2	IND	*R. bursa*	M	6
			1209	33.7	IND	*R. bursa*	F	6
			1210	33.5	IND	*R. bursa*	M	6
			1211	32.9	IND	*Hyalomma marginatum*	M	6
			1212	34.7	40	*R. bursa*	M	6
			1213	33.2	IND	*Hyalomma marginatum*	F	6
			1214	33.1	IND	*R. bursa*	F	6
			1215	33.8	IND	*R. bursa*	F	6
			1218	34.2	IND	*Hyalomma marginatum*	M	4
			1219	35	IND	*Hyalomma marginatum*	F	6
			1220	35.1	IND	*Hyalomma marginatum*	M	3
			1221	34.1	IND	*R. bursa*	M	6
			1222	36.5	IND	*R. bursa*	M	6
			1223	35.7	IND	*Rhipicephalus sanguineus*	M	1
			1224	37.5	IND	*R. bursa*	F	1
Total			24 pools					119

*Pools of ticks were tested with quantitative reverse transcription PCR of the L and S segments of CCHFV RNA. A total of 24 pools comprising 119 ticks were tested CCHFV-positive from 5 animals. CCHFV, Crimean-Congo hemorrhagic fever virus; Ct, cycle threshold; ID, identification; IND, indeterminate (weakly positive); L, large; S, small.

The obtained S and M segment sequences constituted a monophyletic group belonging to genotype I (Africa 1), whereas the L segment sequence grouped with genotype III strains (Africa 3) (Figure). The sequences of all 3 segments of the CCHFV from Corsica are closely related to 2 sequences from Senegal corresponding to strains identified in the 1970s and likely represent strains reassorted in Senegal. Whether those strains are typical of strains from Senegal or have been circulating in other parts of Africa requires additional investigations.

Our results suggest that CCHFV strains circulating in Corsica and Spain have distinct origins. In Spain, genotype III is the most widespread and is most often detected in *H. lusitanicum* ticks (*2*), a species not yet identified in Corsica. Trans-Saharan migratory birds carrying *H. marginatum* ticks are the most likely source of CCHFV strains entering Corsica (*6*). Examination of the main bird migration routes suggests that 2 different migratory corridors link Spain and Corsica to Africa; mainly, but not exclusively, West Africa for Spain and Central Africa for Corsica (*6*). Those migration routes also could explain the different origin of CCHFV strains circulating in Corsica and in Spain.

Our results provide evidence for established CCHFV circulation in Corsica because detection occurred at 2 distinct sites in the southeastern and central western parts of the island. In addition, our results provide evidence for infection in cattle because multiple CCHFV-positive ticks were found on the same animal. CCHFV detection in feeding ticks is indicative of virus circulation within the cattle population but does not elucidate the role of ticks in virus transmission. Our results must be interpreted by considering previous serologic evidence of CCHFV circulation in cattle in Corsica (*3*), the presence of competent vectors locally (*4*), and recent reports of CCHFV detection in southern mainland France (*10*). The threat of possible continuous expansion and circulation of the virus over Western Europe should not be disregarded. Healthcare professionals and other groups at risk for infection, including hunters and farmers, should be informed about CCHFV circulation in Corsica.

Appendix Additional information on Crimean-Congo hemorrhagic fever virus in ticks collected from cattle, Corsica, France, 2023.
