# Supplementary material for: Crimean-Congo Hemorrhagic Fever Virus in Ticks Collected from Cattle, Corsica, France, 2023
Source: Emerg Infect Dis. 2024 May;30(5):1036–9. doi: 10.3201/eid3005.231742 (PMC11060454; doi:10.3201/eid3005.231742)
Supplement: Appendix — Additional information on Crimean-Congo hemorrhagic fever virus in ticks collected from cattle, Corsica, France, 2023. [file 23-1742-Techapp-s1.pdf]

Article DOI: https://doi.org/10.3201/eid3005.231742

EID cannot ensure accessibility for supplementary materials supplied by authors. Readers who have difficulty accessing supplementary content should contact the authors for assistance.

Crimean-Congo Hemorrhagic Fever Virus in Ticks Collected from Cattle, Corsica, France, 2023

Appendix

Appendix Table 1. System 1 for small (S) segment

Name	Sequence	Couple	Position
CrCon1 S	RWA-AYG-GRC-TTR-TGG-AYA-CYT-TCA-C-	Couple 1	123–147
CrCon1 R	TRG-CAA-GRC-CKG-TWG-CRA-CWA-GWG-C		764–740
CriCon2 S	ART-GGA-GRA-ARG-AYA-TWG-GYT-TYC-G	Couple 2	450–474
CriCon2 R	CYTTGA-YRA-AYT-CYC-TRC-ACC-ABT-C		674–650

Appendix Table 2. System 2 for small (S) segment

Primer name	Sequence (5′-3′)	Couple	Amplification size, bp
GP_CCHF_seg S_1S	TCTCAAAGAAACACGTGCCGC	Couple 1	472
GP-CCHF-segS-472R	GCTGTGTTTGCATTGACACGG		
GP-CCHF-segS-444S	GCCAATTACCAACARGCTGC	Couple 2	463
GP-CCHF-segS-907R	AGAGTTCCTGGGCCTTAGTG		
GP-CCHF-segS-845S	CATAAGGACGAAGTTGACAGG	Couple 3	413
GP-CCHF-segS-1258R	TGAGCCCTGGGCTGCATCG		
GP-CCHF-segS-1204S	GCAGAATTAGTGAGATGGGTG	Couple 4	485
GP_CCHF_seg S_1689R	CGCACAGCCCTTTAAGTRTTT		

Appendix Table 3. System 3 for small (S) segment

Primer name	Sequence (5′-3′)	Couple	Amplification size, bp
GP_CCHF_seg S_1S	TCTCAAAGAAACACGTGCCGC	Couple 1	1,689
GP_CCHF_seg S_1689R	CGCACAGCCCTTTAAGTRTTT		

Appendix Table 4. System 4 for medium (M) segment

Primer name	Sequence (5′-3′)	Couple	Amplification size, bp
GP-CCHF-segM-1Rbis	CTMTGYGAGAACAGTGCCWC	Couple 1	351
GP-CCHF-segM-351R	CTGGATTCACCAGAACCACTG		
GP-CCHF-segM-264S	CAATGTCTGTGCTGGAGTCAT	Couple 2	404
GP-CCHF-segM-668R	TGGTTGTGGTCATTACTGATGC		
GP-CCHF-segM-611S	GCACATCATCTCTCCAGAAG	Couple 3	450
GP-CCHF-segM-1061R	CTTGAACAGGCATGCAATGAC		
GP-CCHF-segM-987S	GCAAGATACTGAAACTCCTACA	Couple 4	476
GP-CCHF-segM-1463R	CATGGCAGTTTGAAAGATTAAC		
GP-CCHF-segM-1384S	CAACTGTATAAACTTGAAAGTGTC	Couple 5	479
GP-CCHF-segM-1863R	CTTTGATCCACTATTGTGGAGC		
GP-CCHF-segM-1842Sbis	AGCTATGGTGGKCCTGGYGARA	Couple 6	490
GP-CCHF-segM-2332R	AGTGTCCCGATGACAATTAGC		
GP-CCHF-segM-2296S	CATCACATGTGTAGTGTGCAAG	Couple 7	434
GP-CCHF-segM-2730R	CTCATTTGATAGACTTCAACTG		
GP-CCHF-segM-2692S	GTCACCAGTTCAGTCCGCA	Couple 8	469
GP-CCHF-segM-3161R	GTCGACAGCAGAGGTACTAAC		
GP-CCHF-segM-3084S	GCAACAGGGCTACTTTCATCA	Couple 9	403
GP-CCHF-segM-3487R	CCTGAAACTAAGATCTTGTTGC		
GP-CCHF-segM-3459S	CTTAGCTGGAGTTCAGTTGAAC	Couple 10	305
GP-CCHF-segM-3764Rbis	CGCCAGTTCCTCGAGTGCGGC		

Appendix Table 5. System 5 for medium (M) segment

Primer name	Sequence (5′-3′)	Couple	Amplification size, bp
GP_CCHF_seg M_1S	TCTCAAAGAAATACTTGCGGCAC	Couple 1	1,958
GP_CCHF_seg M_1959R	CCTGTGGCACTGTTTTCGCA		
GP_CCHF_seg M_1842S	AACTATGGTGGYCCRGGTGAYA	Couple 2	1,922
GP_CCHF_seg M_3764R	CTCCAGTTTCTTGARTGRGGC		
GP_CCHF_seg M_3711S	GAYTGCCCGGAAAGRTGTGG	Couple 3	1,855
GP_CCHF_seg M_5566R	TCTCAAAGATATAGTGGCGGC		
GP_CCHF_seg L_12211R	CCCCACACCCCAAAATAATAA		

Appendix Table 6. System 6 for large (L) segment

Primer name	Sequence (5′-3′)	Couple	Amplification size, bp
GP_CCHF_seg L_1S	TCTCAAAGATATCAATCCCCCC	Couple 1	415
GP-CCHF-segL-415S	GGCTAAAGAAATGGGCATTACC		
GP-CCHF-segL-459R	CTGCTTCCACTTCATCACTGG	Couple 2	405
GP-CCHF-segL-864R	GTGTCAGCTGATATCTCAACAC		
GP-CCHF-segL-819S	CCAGAGTCGGTAGAGTCTTG	Couple 3	480
GP-CCHF-segL-1299R	CACTGCAGACAGTTGCTAAGG		
GP-CCHF-segL-1251S	TTCTCGGCAACCTGGGAAATG	Couple 4	238
GP_CCHF_seg L_1489R	GAGATCAGCTATCTCCCTGTG		

Appendix Table 7. System 7 for large (L) segment

Primer name	Sequence (5′-3′)
GP-CCHF-segL-3732s	CTGTGCCAAGATCTTGGCTC
GP-CCHF-segL-4011R	GTGTCTACAATTCCCTCATAAG

Appendix Table 8. System 8 for large (L) segment

Primer name	Sequence (5′-3′)	Couple	Amplification size, bp
GP_CCHF_seg L_1S	TCTCAAAGATATCAATCCCCCC	Couple 1	1,488
GP_CCHF_seg L_1489R	GAGATCAGCTATCTCCCTGTG		
GP_CCHF_seg L_1259S	GYAACCTAGGAAATGAACTGTTG	Couple 2	1,708
GP_CCHF_seg L_2967R	CTTTGCGCATWGCCTGTTCC		
GP_CCHF_seg L_2809S	GTTGTTGGAGCYATAAGTACTC	Couple 3	1,605
GP_CCHF_seg L_4414R	GGARTGATTTTCAATGTCTTG		
GP_CCHF_seg L_4298S	AGGAGRCAAGCTGTCCTTGG	Couple 4	1,888
GP_CCHF_seg L_6178R	CTTTGACARTTCCAGRTGCTG		
GP_CCHF_seg L_6022S	CTCAACGAGCAACAAGATGAAC	Couple 5	1,584
GP_CCHF_seg L_7606R	CATGYTCAACATGTACTGTTGTC		
GP_CCHF_seg L_7491S	GYTACAACCATATGGGTCAGG	Couple 6	1,808
GP_CCHF_seg L_9299R	GCAGGTCTAGACTCAACTATTC		
GP_CCHF_seg L_9077S	CTCACTGGTTGGACACCTTTC	Couple 7	1,547
GP_CCHF_seg L_10624R	CTRTCTGTAGAGCAGTCMAC		
GP_CCHF_seg L_10253S	GTGARACTGAAAGRCAAGTGC	Couple 8	1,958
GP_CCHF_seg L_12211R	CCCCACACCCCAAAATAATAA		
